# Altering gait by way of stimulation of the plantar surface of the foot: the immediate effect of wearing textured insoles in older fallers

**DOI:** 10.1186/1757-1146-5-S1-O21

**Published:** 2012-04-10

**Authors:** Anna L Hatton, John Dixon, Keith Rome, Julia L Newton, Denis J Martin

**Affiliations:** 1 Department of Physiotherapy, The Princess Alexandra Hospital, Brisbane, Queensland 4102, Australia; 2 Health and Social Care Institute, Teesside University, Middlesbrough, Teesside, TS1 3BA, UK; 3 Health & Rehabilitation Research Institute, AUT University, Auckland 1020, New Zealand; 4 Institute for Ageing and Health, University of Newcastle, Newcastle-upon-Tyne, NE1 4LP, UK

## Background

Textured surfaces and shoe insoles can alter standing balance in healthy older adults [1,2] by way of enhanced plantar tactile stimulation. However, it remains unknown whether textured insoles have a similar effect on dynamic balance performance during walking in older people prone to falling. This study explored the immediate effect of textured insoles on gait measurements in older fallers.

## Materials and methods

26 older adults (19 women; mean [1SD] age 79.0 [7.1] years) with a self-reported history of ≥ 2 falls in the previous year, conducted tests of level-ground walking over 10m (GaitRITE system), under two conditions: wearing (in their usual footwear) textured insoles and smooth (control) insoles. Gait measurements included velocity, cadence, step length, stride length, base of support, step time, cycle time, swing time, stance time, and single- and double-limb support times.

## Results

Paired-samples t-tests showed significant reductions in gait velocity (*P*=0.016) and stride length (left *P*=0.028, right *P*=0.043) when wearing textured insoles. Mean (95% CI) differences were: gait velocity -4.20 (-7.55 to -0.85) cm.s^-1^, left stride length -2.92 (-5.49 to -0.34) cm, right stride length -2.87 (-5.64 to -0.09) cm. No significant differences were found for the other gait measures.

## Conclusions

Stimulating the plantar surface of the foot by way of wearing this type of textured insole causes an immediate effect - in this case a slower, more cautious gait in older fallers. Further work is required to determine how textured insoles can be used to improve gait in older fallers.
